# Adipose cellularity as a measurement of long-term changes in body weight: a Swedish cohort study spanning 1988–2016

**DOI:** 10.1016/j.eclinm.2025.103165

**Published:** 2025-03-29

**Authors:** Peter Arner, Thorkild I.A. Sørensen, Daniel P. Andersson

**Affiliations:** a Department of Medicine-H7 at Karolinska Institutet, C2:94 Karolinska University Hospital Huddinge, 14186, Stockholm, Sweden; b Novo Nordisk Foundation Center for Basic Metabolic Research and Department of Public Health Sciences, University of Copenhagen, Blegdamsvej 3B, Copenhagen, 2200N, Denmark; c Center for Childhood Health, Islands Brygge 41, Copenhagen, 2300S, Denmark; d Department of Endocrinology, C2:94 Karolinska University Hospital Huddinge, 14186, Stockholm, Sweden

**Keywords:** Fat cells, Morphology, Weight loss, Weight gain, Adipocyte number, Adipocyte size

## Abstract

**Background:**

Adipocyte size and number (cellularity) determine the adipose mass and may relate to long-term body weight changes.

**Methods:**

We investigated 1014 healthy participants at Karolinska Institutet in Sweden 1988–2016 for body weight and size/number of subcutaneous adipocytes, and 273 for visceral adipocyte size. We measured body weight on 281 subjects about 16 years later. We analysed the association of baseline adipocyte size and number with body weight changes by linear regression including relevant co-factors, and the associations of cellularity (low or high number of either large or small adipocytes) regarding body weight changes by analysis of variance.

**Findings:**

Subcutaneous adipocyte size and number and visceral adipocyte size showed strong relationships with body weight changes irrespective of its mode of expression (adjusted r^2^ ≥0·15). The relationships were significant (p ≤ 0·027) independent of co-factors (age, sex, initial body weight or height, body fat, obesity, nicotine use, physical activity, and observation time). Interventions (lifestyle change or bariatric surgery) did not influence the associations (p = 0·86). A low or high number of large adipocytes associated with body weight loss, whereas a low or high number of small cells associated with weight stability or weight gain.

**Interpretation:**

Adipose cellularity is associated with long-term changes in body weight, following interventions to decrease body weight. Patients with a high number of large fat cells experienced the most pronounced weight reduction.

**Funding:**

The Stockholm County Council (963296, 994175, 986118), the 10.13039/501100018713Center for Innovative Medicine at 10.13039/501100004047Karolinska Institutet (986109) and the 10.13039/501100007687Swedish Society of Medicine (1001156). None of the funding sources had any involvement in the study.

**Research in context**

**Evidence before this study**

Fat cell size and number determine the mass of adipose tissue and can be classified into four categories of adipose cellularity, namely a high or low number of either small or large fat cells. Irrespective of the total amount of adipose tissue, the cellularity is associated in cross-sectional studies with cardiometabolic phenotypes and in longitudinal studies with future risk of developing type 2 diabetes.

**Added value of this study**

To our knowledge, this is the first study examining the relationship between adipose cellularity and body weight changes over time. Subjects with a low or high number of large fat cells were prone to decrease their weight, whereas those with a low or high number of small fat cells experienced weight gain or had stable body weight. This was independent of initial body weight or height, age, sex and interventions with bariatric surgery or a change in lifestyle.

**Implications of all the available evidence**

Adipose cellularity is of pathophysiological importance for cardiometabolic conditions. It may be assessed in patients with overweight or obesity to select those who are most suitable for body weight reducing therapy, which seems less effective when adipose tissue is composed of few or many small fat cells.

## Introduction

Changes in amount of body fat mass contribute to increase or decrease in body weight.^1^ The fat mass amount is determined mainly by the size and number of adipocytes in adipose tissue. The relationship between size and number of cells, often called cellularity as herein, allows distinction between four different types of adipose cellularity, namely a high or low number of either large or small adipocytes. Adipose cellularity is in a highly dynamic state as reviewed.^1^ During infancy and adolescence, the fat mass expands by increasing size and number of its adipocytes. Despite a rather rapid adipocyte turnover in adulthood^2^ the number of adipocytes is relatively stable during adult aging, and does not change during weight loss, but can increase during weight gain.^1^ On the other hand, adipocyte size changes in parallel with increase or decrease in body weight.^1^ The cellularity is dependent of the turnover of adipocytes, being slower with large cells than with small cells.^3^ Numerous cross-sectional studies have been published regarding the clinical impact of adipose cellularity as reviewed.^4^^,^^5^ The different forms of cellularity are not merely a reflection of the actual amount of body fat; people with or without obesity can have few large or many small subcutaneous adipocytes.^3^ The subcutaneous region represents about 80% of the total adipose tissue mass, with the remainder distributed throughout the body with a considerable part in the abdominal visceral area.^6^

A yet unanswered and major question is whether adipose cellularity plays a role for future long-term changes in body weight. Intuitively having a low number of small adipocytes makes room for increase in their size and number, and thereby for weight gain, whereas having many large cells might facilitate a decrease in their size without changing in number and thereby promote weight loss. This question is also clinically important because pharmacotherapy and lifestyle modification may alter the cellularity as discussed.^5^ Furthermore, adipose cellularity (adipocyte size in relation to number) could possibly predict future weight gain and/or the outcome of therapies targeting excess fat mass.^7^ A couple of previous investigations suggested that the cellularity influences later body weight changes. Thus, a small and short-term study on patients with obesity demonstrated that baseline subcutaneous adipose cellularity relates to the body weight loss following energy-restricted diet.^8^ Another study on Pima Indians showed that initial large adipocyte size was moderately associated with later increase in fat mass.^9^

In the present study we investigated if the number and/or size of adipocytes could predict future long-term changes in body weight. Two hundred eighty-two participants had their baseline abdominal subcutaneous adipose tissue cellularity examined and related to subsequent changes in body weight after a mean observation time or 16 years. In a subgroup the size of visceral adipocytes were also related to body weight changes over time.

## Methods

### Patients

During 1988–2016, 1014 participants were recruited by local advertising or from the departments of Surgery and Medicine at Karolinska University Hospital, Huddinge, Sweden for measurement of subcutaneous adipose cellularity and clinical characteristics. About 95% were of Scandinavian origin. All lived in the Stockholm County area. As regards inclusion criteria we enrolled everyone who self-reported to be in general good health and was willing to undergo the examinations. As regards exclusion criteria we excluded participants with ongoing acute episode of severe disease, which were never used. The participants have been included in several cross-sectional studies as exemplified.^3^^,^^10^^,^^11^ They came to the laboratory in the morning after an overnight fast. At that time body weight had been stable for at least 6 months according to self-report (<±2 kg change). Body weight, height and circumferences of hip and waist were determined. A venous blood sample was obtained for routine clinical chemistry measurements, and total body fat was measured by impedance (BodyStat Quadscan 4000, Isle of Man, British Isles). In 200 individuals, impedance measurements were not performed and instead body fat was estimated by a formula based on body mass index (BMI), age and sex.^12^ We have in the supplement of a previous study shown that the values for fat mass were very similar when comparing measures using impedance, the formula above or the “gold standard” dual x-ray absorptiometry.^3^ We used a questionnaire to establish sex (all reported being males or females as at birth) and evaluate overall physical activity according to a four graded scale. This gives a validated classification of the patients being sedentary or active.^11^ Finally, a specimen of subcutaneous adipose tissue was obtained by needle aspiration from the subcutaneous paraumbilical area. Within a month after the examination at our laboratory, 273 of the participants underwent laparoscopic bariatric surgery for obesity (or cholecystectomy) and a visceral (omental) adipose tissue specimen was obtained at the beginning of surgery. During 2018–2021 all investigated participants were contacted for a clinical re-examination at their health care centre plus requested to fill in a health questionnaire. Two hundred eighty-one participants responded, all with the questionnaires. The mean observation time was 16 years (range 5–28 years). Since actual body weight was not always recorded, we used the questionnaire information for body weight. In participants with data for examined as well as self-reported weight there was an excellent correlation between self-reported weight and actual body weight when measured at the health care centre (adjusted r = 0·995, slope = 1·006 by linear regression). The questionnaire included information about any intervention to reduce body weight. Forty-eight had changed diet or physical exercise habits (lifestyle), 69 had undergone bariatric surgery and 164 reported no intervention. None had used anti-obesity pharmacotherapy during the observation period. One-hundred eighty-five visited the health care centre and 96 only gave information using the questionnaire. A flow chart of the first and second examination is shown in Fig. S1 containing information about the key measures.

### Ethics

The data collection is based on data from several previous projects, and all have been approved by the Regional Ethics Committee in Stockholm, Sweden (Ethical grant numbers; 114/92, 200/98, 117/99, 167/02, 592/03, 534/03, 163/03, 2008/1010-31/3, 2011/1102 31/1, 2016/2583 and 31/1, 2018/809-31). The ethics permit from 2018 allowed us to retrospectively analyze all clinical and adipose data from previously approved applications. The baseline experiments were explained in detail to each participant and written informed consent was obtained from each participant. For four patients aged 16–17 years, informed consent was obtained from themselves as well as from their parents. The local committee on ethics approved each baseline study and the present follow-up study, which was mentioned in the invitation letter to the participants.

### Adipose tissue examinations

Abdominal subcutaneous adipose tissue mass was determined using a validated algorithm.^10^ The equation is: kg of adipose tissue = −3·317 + (waist-to-hip ratio × 0·889) + gender (1 for men and 2 for women) × 0·394—age in years × 0·012 + total body fat mass in kg × 0·015 + waist circumference in cm × 0·051. The regression line for comparison with actual measure has slope 1·02 and intercept 0·01.^10^ Fat cell size and number were determined as described.^10^ In brief, adipocytes were isolated by collagenase treatment. The diameter of 100 cells was determined with a light microscope and used for calculation of mean adipocyte weight and size with established equations.^13^ There is no improvement by measuring >100 cell diameters.^14^ Some laboratories show bimodal distribution of adipocyte diameters.^15^ However, we only found a unimodal distribution.^16^ Adipocyte number was obtained by dividing the weight of abdominal subcutaneous tissue with the mean adipocyte weight.

### Characterization of adipose cellularity

There is no consensus how to characterize adipose cellularity. It is possible to only use fat cell size and compare it with the fat mass.^3^ However, this relationship is curve linear and may require complicated statistical procedures when groups are compared.^2^ A more straight-forward way is to use both size and number to characterise cellularity.^10^ We used the latter procedure to phenotype abdominal subcutaneous adipose tissue cellularity in the whole study group examined at baseline. Median values for size and number were separately defined. The median values were 681 pL and 0.336 × 10^9^ cells, respectively. Subjects with values above median for cell size or number were defined as having large/many fat cells. Those with values below the median were defined as few/small cells. Complete information was available in 965 participants and generated the following cellularity phenotypes, many large cells (n = 327), many small cells (n = 152), few large cells (n = 167) and few small cells (n = 319). The cellularity classification was maintained in the re-investigated participants.

### Statistics

Analyses were performed in JMP Version 16.1.0 (SAS, Institute Inc., Buckinghamshire, UK) and Graphpad version 10.0.2 (Graphpad Software, Boston, USA). Values for clinical and adipose variables are expressed as mean and range in text or tables and as box plots or individually in figures. The major outcome variable was changes over time in body weight parameters. The major independent variables were initial adipocyte size and number. The primary investigation was the relationship between these independent and dependent variables using linear regression analysis. The influence of co-factors on the mentioned relationships was investigated by analysis of covariance (ANCOVA) or by linear regression in subgroups. We used Bonferroni correction in the linear regression analysis when investigating subgroups. The influencing factors on body weight and/or adipose cellularity parameters were age, sex, initial body fat or body weight, height, physical activity, nicotine use, initial presence of obesity (BMI ≥30 kg/m^2^) and observation time.^17^, ^18^, ^19^, ^20^, ^21^ The second comparison was between different forms of initial adipose cellularity and changes in body weight parameters over time using analysis of variance (ANOVA). If appropriate the values for the four different cellularity forms were subsequently compared between each other using unpaired t-test or examined for difference from zero using one-group t-test. Additional tertiary tests were between groups by Fisher's exact test, or within a group at first and second examination using paired t-test. Prior to the re-investigation was completed we made a statistical power calculation assuming a 30% compliance, which we considered to be acceptable bearing in mind the long period between initial investigation and follow-up. In single regression we could detect a medium size effect (beta-coefficient = 0·15) at p = 0·05 with 80% power including 50 participants. This suggest that we could make valid statistical analysis in small subgroups.

### Role of funding

None of the funders had any role in study design, data collection, data analyses, interpretation, or writing of the report.

## Results

The clinical characteristics for those followed up and those not followed up are recorded in Table 1. There were only some minor differences between those that were re-investigated and those that were not re-investigated. A flowchart for the study population is found in Fig. S1.

**Table 1:** Characteristics of patients who were re-investigated or did not participate in re-investigation.

Phenotype	Re-investigated (n = 282)		Not re-investigated (n = 732)		p-value
	Mean	Range	Mean	Range	
Sex (males/females)	86/196		199/533		0·31
Age (years)	44	21–72	41	16–79	<0·0001
Body weight (kg)	91	48–164	95	48–163	0·02
Height (cm)	170	148–195	170	145–199	0·48
Waist-to-hip (ratio)	0·933	0·699–1·121	0·942	0·705–1·209	0·12
Body mass index (kg/m^2^)	32	18–53	33	18–63	0·02
Body fat (% of total weight)	41	14–75	44	8–86	0·005
Obesity/not obesity	146/136		411/318		0·20
Nicotine use (yes/no)	48/229		191/510		0·0005
Sedentary/physically active	71/175		205/385		0·10
P-glucose (mmol/l)	5·5	3·4–12·9	5·5	3·6–20·9	0·89
S-insulin (mU/l)	10·6	1·6–42·1	12·3	1·1–60·0	0·001
P-triglycerides (mmol/l)	1·5	0·3–20·0	1·5	0·0–21·7	0·63
P-total cholesterol (mmol/l)	5·0	2·7–9·8	5·0	2·5–12·6	0·76
P-HDL cholesterol (mmol/l)	1·3	0·5–2·4	1·3	0·5–2·9	0·04
Abdominal subcutaneous adipocyte number ×10^9^	3·2	0·4–6·5	3·5	0·01–15·7	0·003
Abdominal subcutaneous adipocyte volume (picolitres)	667	144–1379	689	72–1452	0·21

Values were compared by unpaired t-test or Fisher's exact test. S, fasting serum; P, fasting plasma.

**Fig. 1:**
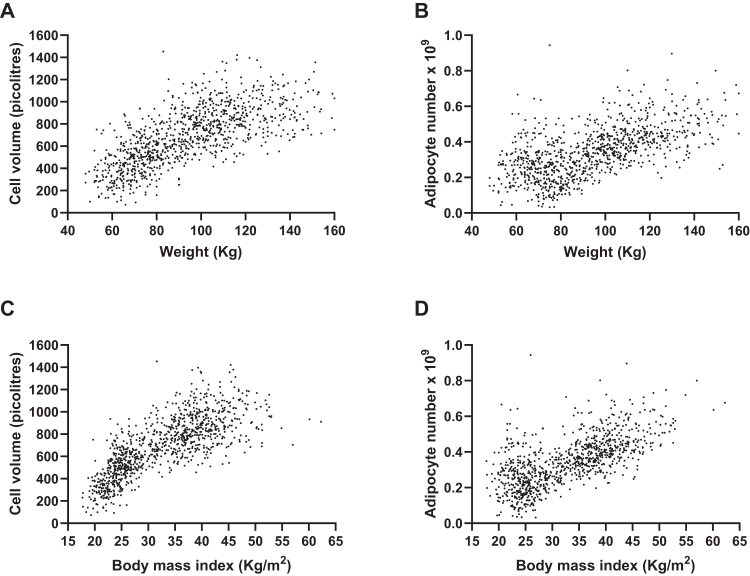
Relationship between body weight (A, B) or body mass index (C, D) and abdominal adipocyte size (A, C) or number (B, D) in all subjects investigated at baseline. Adipocyte size or number was measured in 1004 and 965 subjects, respectively. One subject with 1.6 × 10^9^ adipocytes is not shown in the graph.

Fig. 1 depicts the distribution of abdominal subcutaneous adipocyte size and number in all subjects investigated at baseline irrespective of follow-up. The cohort displays a broad range of body weight (about 60–130 kg) and BMI (about 20–40 kg/m^2^). As expected, size and number increased by increasing body weight or BMI. However, over the broad range of body weight/BMI, the adipose tissue could have any combination of fat cell size and number.

**Fig. 2:**
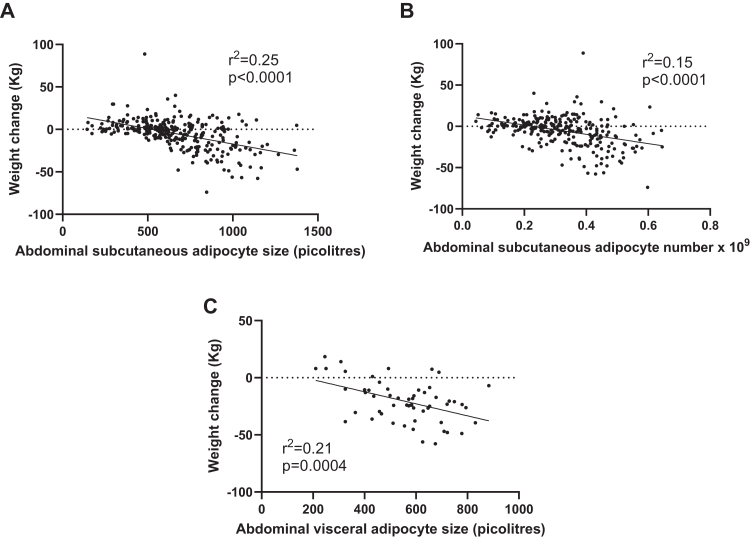
Relationship between abdominal subcutaneous adipocyte size (A) or number (B) and omental adipocyte size (C) on the one hand and kg body weight changes between first and second examination on the other hand. Linear regression analysis was used. The quotas of subjects for abdominal subcutaneous adipocyte size and number are 282 and 274, respectively. The number for omental adipocyte size is 56.

**Fig. S2:**
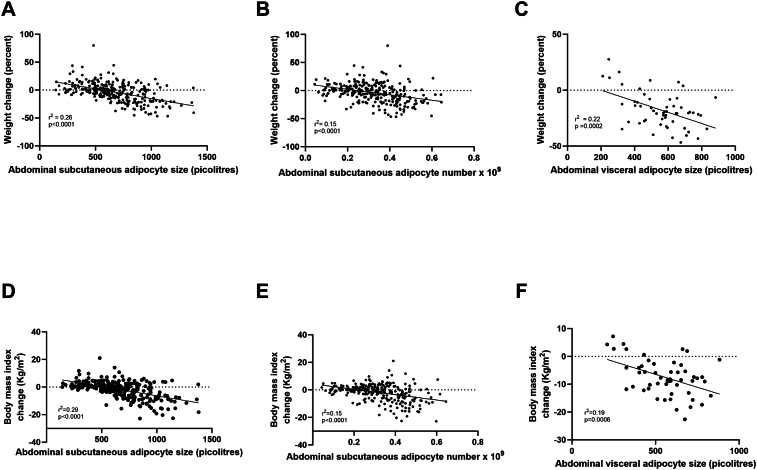
Relationship between abdominal subcutaneous fat cell size (A, B), visceral fat cell size (C, E) or abdominal subcutaneous fat cell number (D, E) on the one hand, and % body weight change (A-C) or body mass index change (D-F) on the other hand. Linear regression was used. Fat cell size and number were obtained at the first examination. Difference between body weight at first and second examination were calculated as % of initial weight. Body mass index at second minus first examination was calculated. The number of subjects is 281, 273, 55, 265, 257, and 53 in A, B, C, D, and F, respectively.

Fig. 2 depicts the findings with adipocyte size and number by themselves in relation to changes over time in kg body weight. In abdominal subcutaneous adipose tissue both the number and size of adipocytes showed a strong inverse relationship with changes in body weight over time, which was also true for visceral adipocyte size (we had no information about visceral adipocyte number). The inverse relationship means that the larger the cells or cell number the more pronounced was body weight loss and the other way around. On average, variations in initial fat cell size and number explained 25% and 15%, respectively, of the between patient variations in body weight changes over time. Similar results were obtained if % body weight changes or changes in BMI were used as outcome variable (Fig. S2). For height there was a small decrease (average 0·5 cm) over time (p < 0·0001), but the changes in height and body weight were not correlated (r = 0·05; p = 0·38). Because of the latter findings in combination with those in Fig. 1 and Fig. S2, we considered changes in kg body weight was the most physiological outcome variable and used it in the subsequent analyses unless otherwise stated.

We next investigated if co-factors influenced the relationship between adipocyte size or number and body weight changes using the subcutaneous data. Several ANCOVA models were created for adipocyte number or size together with either of the following co-factors; sex initial age, observation time, and initial body weight, height, BMI or body fat percent (Table 2). We did not use a comprehensive multiple regression model putting all co-factors together for the following reasons. Many of the co-factors were dependent of each other and our sample was not large enough to make analyses of the individual contributions. Furthermore, multiple regression does not allow us to consider interactions between factors in contrast to ANCOVA. In the model in Table 2 initial BMI, body fat or body weight interacted significantly with fat cell size or number in the contribution to changes in body weight over time. However, in all models except one, adipocyte size or number contributed significantly and independently to the variations over time in kg body weight (p ≤ 0·027). The exception was that fat cell number was not a significant regressor in the presence of body fat. There was also a slight interaction between sex and fat cell size in the contribution to change in body weight, but the separate effect of size was prominent (F = 44). Adipocyte size contributed independently to body weight variations in the presence of body fat. We further investigated the independent roles of subcutaneous adipocyte size and number by performing linear regression in 8 subgroups (Table S1). Size or number showed a similar negative relationship with body weight changes as for the whole group in men and women, in sedentary and physically active people, in participants using nicotine and not, and in those with or without initial obesity. When correcting for multiple comparison the threshold value for statistical significance would be p < 0·05/8. Only one out of 14 nominally significant p-values was above the adjusted p-value.

**Table 2:** Influence of independent factors on the relationship between size or number of abdominal subcutaneous fat cells and body weight changes.

Independent variable	Adipocyte size (picolitres)		Adipocyte number	
	F-value	p-value	F-value	p-value
Size or number + sex				
Size/number	44·4	<0·0001	28·8	<0·0001
Sex (male or female)	4·3	0·04	0·25	0·61
Size/number × sex	0·6	0·43	1·3	0·26
Size or number + initial age				
Size/number	95·5	<0·0001	66·6	<0·0001
Initial age (years)	3·7	0·053	17·4	<0·0001
Size/number × age	0·13	0·71	0·2	0·64
Size or number + observation time				
Size/number	100·8	<0·0001	51·4	<0·0001
Observation time (years)	7·3	0·007	5·2	0·02
Size/number × time	0·02	0·88	0·03	0·87
Size or number + initial body weight				
Size/number	19·8	<0·0001	6·2	0·01
Initial body weight (kg)	17·8	<0·0001	30·9	<0·0001
Size/number × body weight	16·8	<0·0001	9·3	0·0025
Size or number + initial height				
Size/number	77·4	<0·0001	41·3	<0·0001
Initial height (cm)	3·5	0·06	7·1	0·008
Size/number × initial height	0·4	0·52	3·1	0·08
Size or number + body fat percent				
Size/number	5·4	0·02	0·0001	0·99
Body fat percent	35·8	<0·0001	66·7	<0·0001
Size/number × body fat percent	20·0	<0·0001	18·5	<0·0001
Size or number + body mass index				
Size/number	13.5	0.0003	5.0	0.027
Body mass index (kg/m^2^)	0.1	0.76	2.8	0.09
Size/number × body mass index	17.8	<0.0001	4.8	0.029

Size or number was investigated together with sex, initial age, observation time, initial body weight, initial height, initial % body fat or initial body mass index using separate analysis of covariance models. The contribution of the individual independent factors to body weight changes and the interaction between size or number and each of the other independent factors was analysed. The number of patients in the models was 270–282 for size or number.

**Fig. 3:**
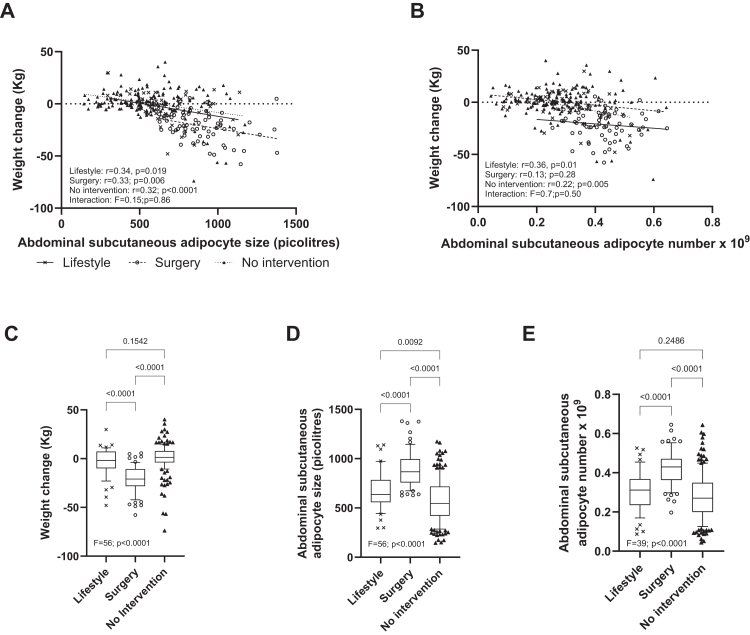
Findings with body weight intervention (lifestyle with diet/exercise or bariatric surgery) and no intervention. The relationship between initial abdominal subcutaneous adipocyte size (A) or number (B) and body weight changes was investigated in the three conditions separately by simple linear regression and the interaction between conditions and adipocyte size was assessed by analysis of covariance. (C–E) The influence of condition leading to body weight changes and initial abdominal subcutaneous adipocyte size was investigated by analysis of variance followed by Tukey's multiple comparisons test. In A, C and D the number of subjects are 48, 69 and 164 for lifestyle, surgery and no intervention, respectively. For adipocyte number the corresponding number of individuals were 48, 67 and 158 in B and E.

We also investigated the influence of interventions to decrease body weight (Fig. 3). Participants were grouped by whether they had changed their lifestyle, undergone bariatric surgery (mainly gastric bypass or banding), or not been subjected to an intervention. None had tried pharmacotherapy. Clinically the groups differed considerably. The surgery group had the largest adipocyte sizes as well as the highest number of adipocytes, and the individuals in this group lost, as expected, more in body weight than the other two groups (Fig. 3C–E). Nevertheless, the inverse relationship between adipocyte size and changes in body weight over time (Fig. 3A) was maintained in each subgroup according to linear regression (p ≤ 0·019), and similar in all three groups according to ANCOVA (p = 0·86). As recorded in Fig. 3B adipocyte number related inversely to body weight changes in the lifestyle (p = 0·01) and non-intervention groups (p = 0·005), but not in the surgery group (p = 0·28) using linear regression analysis. On the other hand, the relationship between fat cell number and body weight changes was not statistically different between the three groups according to ANCOVA (p = 0·50). Five nominally significant p-values were recorded in Fig. 3A and B. After correction for multiple comparison (new threshold was p < 0·05/3) the relationship between fat cell size and body weight change was border line statistically significant.

**Fig. 4:**
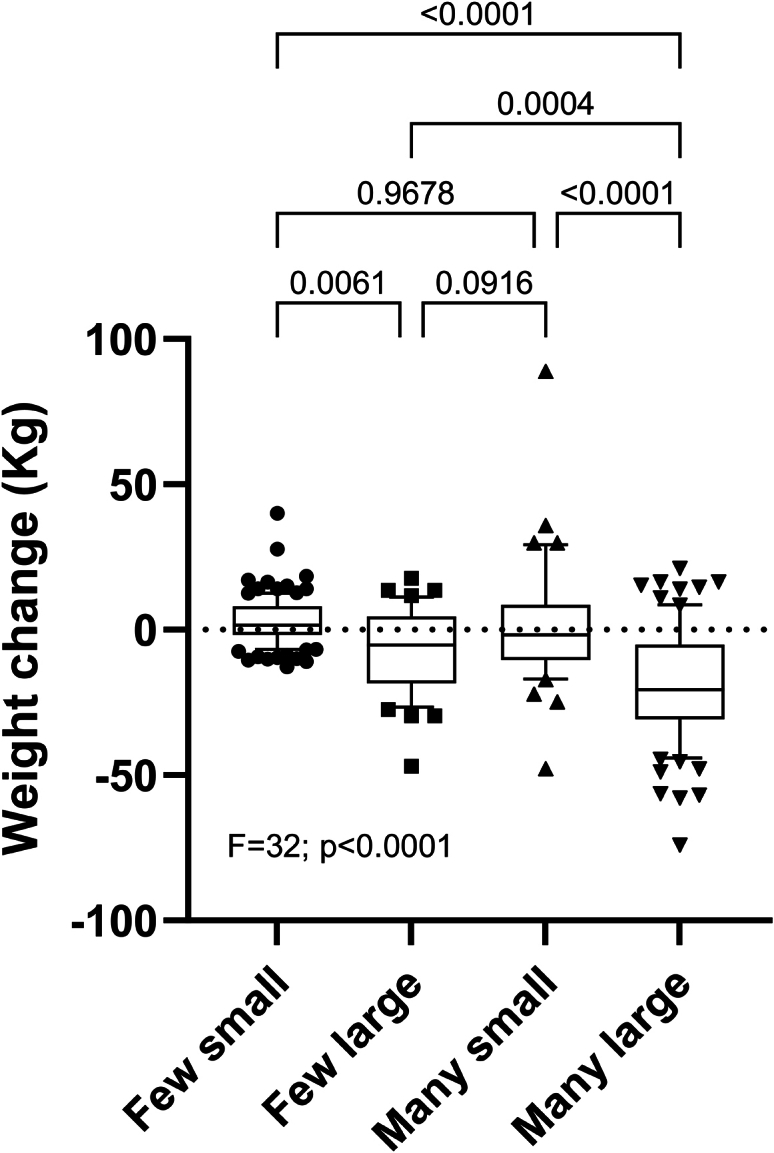
Long term kg body weight changes in different forms of abdominal subcutaneous adipose cellularity. The median value for adipocyte size and number in the whole group examined at baseline was used to create four types of cellularity, namely few small (n = 110) or large (n = 42) adipocytes and many small (n = 41) or large (n = 81) adipocytes. Analysis of variance and Tukey's multiple comparisons test was used in the analyses.

We subsequently investigated adipocyte size and number put together (cellularity) and studied the role of initial abdominal subcutaneous adipose cellularity on body weight changes. For this purpose, we used the specific median values for size and number in the whole group examined at baseline to categorize the participants into four types of cellularity namely having few small -, many small -, few large - or many large cells (Fig. 4). Overall, the effect of different forms of cellularity on body weight changes was significant (p < 0·0001) by ANOVA. The contributions to body weight changes of adipose cellularity, sex, age, observation time and height were put together in an ANCOVA. Cellularity was by far the strongest regressor in this model (F = 33; p < 0·0001). However, the impact of individual types of cellularity differed. According to one group t-test, those with either few or many large cells lost body weight (p = 0·005 and p < 0·0001 respectively). Those with few small cells increased body weight (p = 0·0007) whereas those with many small cells were weight stable (p = 0·68). We also tested the difference between those with many large cells and the other cellularities with un-paired t-test (Fig. 4). The magnitude of weight change was most prominent in those with a high number of large cells.

**Fig. S3:**
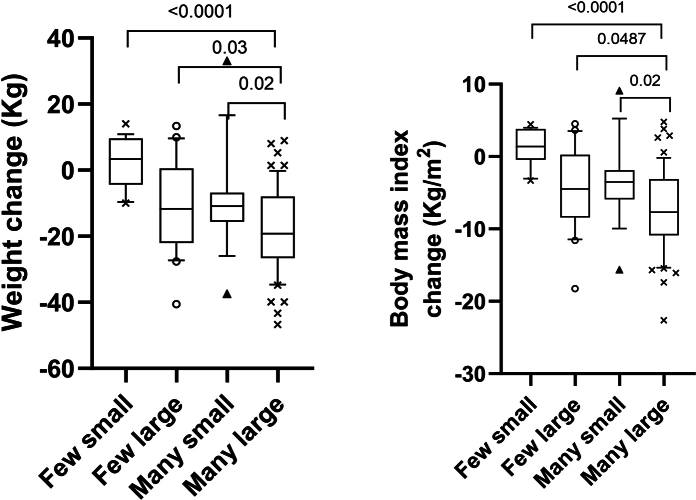
Relationship between adipose cellularity and changes in body weight parameters after intentional intervention for excess body weight. This is a sub analysis of data in Figure 4 excluding subjects who had no intervention for excess body weight. The subjects undergoing lifestyle changes or bariatric surgery were combined. Grouping into four categories of cellularity was done as described in Methods. The numbers of subjects with few small, many small, few large or many large fat cells were 19, 20, 17, and 59, respectively. Body weight and body mass index (BMI) were analyzed at first and second examination and the changes (second – first examination) were calculated as weight change (A), and BMI change (C). The initial comparison of the four groups was made by analysis of variance. Significant differences (p<0.0001) between groups were recorded (F= 12–14). The second comparison was between many large cells and each of the other three types of cellularity by unpaired t-test. The p-value for each comparison is displayed above the compared cellularity.

Finally, the usefulness of adipose cellularity to predict changes in body weight following intervention was analysed by combining lifestyle and bariatric surgery (shown separately in Fig. 3). As expected, there was an overall significant (p < 0·0001) effect of cellularity on body weight changes as judged by ANOVA (Fig. S3A). According to one group comparison body weight was not changed among those with a low number of small cells (p = 0·23) but the other three groups displayed significant weigh loss (p ≤ 0·0125). These changes were most prominent for patients having a high number of large fat cells according to unpaired t-test, being about twice that in participants with low number of large cells or high number of small cells.

## Discussion

Despite numerous studies published over a long period, reviewed here,^1^^,^^4^^,^^5^ the role of adipose cellularity for weight gain, weight loss or weight stability during adulthood is not known. The study showed, as expected,^1^, ^2^, ^3^, ^4^, ^5^ a great inter-individual variability in adipose cellularity at baseline and demonstrates that it plays a hitherto unknown role for long-term changes in body weight. Participants with a high or low number of large fat cells are prone to weight loss whereas those with a high or low number of small adipocytes are weight stable or gain weight.

The statistical power assessment suggested that we could detect physiologically meaningful influence of adipose cellularity in a small number of participants. About 30% of the initially investigated participants participated in the re-investigation. This figure is in line with the follow-up rate of many other and larger studies.^22^, ^23^, ^24^ Importantly, the baseline clinical and adipose cellularity data were rather similar between those who did or did not participate in the re-examination, which suggests that the individuals that were re-examined were representative for the whole group.

When considering adipocyte size and number separately, both seem to be important for future changes in body weight in an inverse manner explaining 25% of the changes in body weight for adipocyte size and 15% for adipocyte number. For both measures body weight decreased progressively by increasing size or number of adipocytes and the other way around. The reverse relationship with adipocyte size and number was not dependent on how the outcome variable was calculated (kg, % body weight change, or delta BMI). It appears to be robust, because initial body weight, height or body fat, age, sex, physical activity status, observation time, and use of nicotine according to multivariate or subgroup analyses did not influence the relationships in important ways except for body fat and adipocyte number. Other factors not examined herein, such as socioeconomic status, diets and alcohol use, might influence the role of adipose cellularity.

We used a common method to measure fat cell size which has been validated thoroughly by us and others.^10^, ^11^, ^12^, ^13^, ^14^^,^^16^ The determination of fat cell number is dependent on the adipose tissue weight and mean fat cell weight and can be obtained by different methods as reviewed.^5^ Nevertheless, values for fat cell number in the body are not much influenced by the use of methods as demonstrated in a previous study where we compared our own data from adults with those obtained with children and adolescents in another laboratory about 20 years earlier.^2^ The early study used total potassium for body fat and measured fat cell size in pieces of subcutaneous adipose tissue whereas we used the formula mentioned in the Method section to determine fat mass and measured adipocyte size in isolated subcutaneous fat cells. Regardless of the marked differences in methodology it was possible to make a valid comparison of the two data sets as shown in Fig. 2 of our previous study.^2^

Our findings deviate from those in a published study of Pima Indians showing a weak positive correlation between initial abdominal subcutaneous adipocyte size and relative increase in total body fat content over time.^9^ There could be many reasons for the divergent results. Preceding body weight or BMI was not available, and observation time was much shorter, about 8 years compared with 16 years in our study. Furthermore, the Pima Indians had a high risk of increasing their body fat mass over time.^9^

Body weight loss can be intentional or non-intentional. The results herein suggest that the role of adipocyte size for future body weight changes was not influenced in an important way if weight loss treatments with lifestyle modification or bariatric surgery (mainly gastric bypass or gastric banding) were compared with no intervention. The same seems to be true for adipocyte number regarding change in lifestyle compared to no intervention.

As discussed here,^4^^,^^25^ there are regional variations in adipocyte size, but they are less likely to be of great importance for the present findings. Thus, we found the same type of inverse relationship to and body weight changes with adipocyte size in subcutaneous and visceral adipose tissue.

The findings in Fig. 4, where the combined effects of size and number (cellularity) were considered, support the notion that adipocyte number and size act in consonance in the putative regulation of future changes in body weight. Regardless if the total number was high or low large cells associated with weight loss, which was most pronounced when many cells were present. On the other hand, many small cells associated with weight stability, and a low number of small cells with some weight gain. This could be a pathophysiological advantage because large fat cells link to a metabolically pernicious phenotype, whereas small cells may protect from such an adverse profile.^3^, ^4^, ^5^ Induced weight loss could have a greater effect when many large fat cells are present than with many small ones, as indicated by a recent study of the outcome of bariatric surgery.^26^

Our findings are not just a reflection of obesity and intervention against body weight. First, the numbers of individuals with or without obesity were about the same (Table 1), Second, the results with size and number of fat cells were not dependent of initial body weight parameters. Although there was an interaction between BMI and fat cell size or number the latter factors remained significant contributors to body weight changes over time (Table 2). Third, the results were similar when the re-investigated were subdivided into no intervention, lifestyle change or treatment with bariatric surgery (Fig. 3).

Numerous factors are involved in the regulation of fat mass and thereby body weight in adults.^27^ Our study suggest that adipose cellularity could play an additional and hitherto unrecognized role. Thus, we propose that adipocyte size, independent of adipose region and adipocyte number (at least in the subcutaneous region), influence long-term body weight changes. Having large cells facilitate both intentional and nonintentional weight loss, most pronounced when there are many of them. Having small cells protects from weight loss and may even promote some weight gain when there are few of them. Hypothetically, subjects with a low number of small adipocytes have reduced possibilities to lose weight by reducing their fat mass so the most physiological way for them is to gain weight by getting more and/or larger adipocytes. The relationships could also be pathophysiologically meaningful, because it is more metabolically favourable to reduce the size of large than small adipocytes during body weight loss. It is less likely that different roles of large versus small adipocytes is due to variations in the turnover of triglyceride in their lipid droplet. Large and small subcutaneous adipocytes have similar triglyceride age, indicative of similar lipid turnover.^28^ Clearly, additional independent and longitudinal studies must test these hypotheses.

Because of the potentially clinically important role of adipose cellularity, there is a need for methods determining adipocyte size usable outside of specialized laboratories. This might be of particular clinical benefit when predicting the outcome of powerful weight reducing programs such as bariatric surgery and incretin therapy. Unfortunately, most of the methods are not suitable for such a purpose, because they necessitate investigation on fresh adipose material and cumbersome cell sizing with manual or semi-automated methods.^4^^,^^5^ However, small adipose pieces, easily obtained by needle aspiration, can be kept in paraffin embedding, and then analysed for adipocyte size with fully automated methods.^29^

Present results and previous studies suggest that determination of abdominal subcutaneous adipose cellularity could be useful in the clinics. Large fat cells associate independent of BMI with risk of developing type 2 diabetes in the future.^30^^,^^31^ Adipose cellularity but not fat cell size per se associates with amelioration of insulin resistance after bariatric surgery for obesity.^32^ The findings in Fig. S3 herein indicate the fat cell cellularity can be useful to predict weight loss after therapeutic intervention. Such treatments may be ineffective in patients having few small fat cells but particularly effective when the patients have a high number of large fat cells to start with.

There are some caveats with the present study. The convenience type of recruitment implied that it was not population based, but even such a study may not be representative for the general population, because people who volunteer for a fat biopsy might be different from those who do not want to participate. To avoid a selection bias, we included all participants who were willing to participate and restricted recruitment to those living in the Stockholm, Sweden area. Ethnicity was rather homogenous because only about 5% of the investigated subjects were of non-Scandinavian origin. However, the possible causal role of adipose cellularity for future changes in body weight cannot be determined with the present data, because of its observational design. Because we only measured body weight at the second examination, we do not know how selective changes in body composition such as in fat, muscle, bone and water content may influence the results.

In summary, adipose cellularity is important for long-term changes in body weight. Having a low or high number of large adipocytes associate with weight loss, whereas having few or many small such cells associate with weight stability or weight gain. Patients with many large fat cells may benefit the most of therapeutic intervention for excess body weight whereas such treatment seem ineffective when adipose tissue is composed of a low number of small fat cells. These notions have to be supported by future large scale prospective analysis.

## Contributors

All authors contributed to the planning of the study and had access to data. PA and DPA analysed the results. All authors had full access to all the data in the study and accepted the final version of the manuscript for submission. PA wrote the first version of the paper. All authors contributed to further writing. All authors read and approved the final version of the manuscript. DPA and PA had access to and verified the underlying data.

## Data sharing statement

All data underlying tables and figures are available upon reasonable request from someone experienced in clinical research.

## Declaration of interests

The authors declare that they have no competing interests.

## Appendix A Supplementary data

Fig. S1 Flow chart for key measures. In total 1014 subjects were initially investigated. For subcutaneous (sc) number data were missed on 49 subjects and for body weight/body mass index (BMI) data were not recorded for 3 subjects. Visceral fat cell size was recorded for a fraction of the initially investigated subjects. On all of them we had data for body weight and/or BMI. A second investigation was performed on 281 subjects. On all of them we had follow up data on body weight and BMI as well as initial data on sc fat cell size. Data on sc fat cell number was missing on 7 subjects but on all remaining ones we had initial and follow up data on body weight/BMI. For the subgroup with initial data on visceral fat cell size we had body weight/BMI data at initial–and follow-up investigation.

## References

[1] Arner P., Rydén M. (2022). Human white adipose tissue: a highly dynamic metabolic organ. J Intern Med.

[2] Spalding K.L., Arner E., Westermark P.O. (2008). Dynamics of fat cell turnover in humans. Nature.

[3] Arner E., Westermark P.O., Spalding K.L. (2010). Adipocyte turnover: relevance to human adipose tissue morphology. Diabetes.

[4] Stenkula K.G., Erlanson-Albertsson C. (2018). Adipose cell size: importance in health and disease. Am J Physiol Regul Integr Comp Physiol.

[5] Ye R.Z., Richard G., Gévry N., Tchernof A., Carpentier A.C. (2022). Fat cell size: measurement methods, pathophysiological origins, and relationships with metabolic dysregulations. Endocr Rev.

[6] Sakers A., De Siqueira M.K., Seale P., Villanueva C.J. (2022). Adipose-tissue plasticity in health and disease. Cell.

[7] Busebee B., Ghusn W., Cifuentes L., Acosta A. (2023). Obesity: a review of pathophysiology and classification. Mayo Clin Proc.

[8] Bosello O., Ostuzzi R., Rossi F.A. (1980). Adipose tissue cellularity and weight reduction forecasting. Am J Clin Nutr.

[9] Frankl J., Piaggi P., Foley J.E., Krakoff J., Votruba S.B. (2017). In Vitro lipolysis is associated with whole-body lipid oxidation and weight gain in humans. Obesity (Silver Spring).

[10] Andersson D.P., Arner E., Hogling D.E., Rydén M., Arner P. (2017). Abdominal subcutaneous adipose tissue cellularity in men and women. Int J Obes (Lond).

[11] Andersson D.P., Kerr A.G., Dahlman I., Rydén M., Arner P. (2023). Relationship between a sedentary lifestyle and adipose insulin resistance. Diabetes.

[12] Gallagher D., Visser M., Sepúlveda D., Pierson R.N., Harris T., Heymsfield S.B. (1996). How useful is body mass index for comparison of body fatness across age, sex, and ethnic groups?. Am J Epidemiol.

[13] Goldrick R.B., McLoughlin G.M. (1970). Lipolysis and lipogenesis from glucose in human fat cells of different sizes. Effects of insulin, epinephrine, and theophylline. J Clin Invest.

[14] Tchoukalova Y.D., Harteneck D.A., Karwoski R.A., Tarara J., Jensen M.D. (2003). A quick, reliable, and automated method for fat cell sizing. J Lipid Res.

[15] McLaughlin T., Sherman A., Tsao P. (2007). Enhanced proportion of small adipose cells in insulin-resistant vs insulin-sensitive obese individuals implicates impaired adipogenesis. Diabetologia.

[16] Hoffstedt J., Andersson D.P., Eriksson Hogling D. (2017). Long-term protective changes in adipose tissue after gastric bypass. Diabetes Care.

[17] Filozof C., Gonzalez C. (2000). Predictors of weight gain: the biological-behavioural debate. Obes Rev.

[18] Stubbs J., Whybrow S., Teixeira P. (2011). Problems in identifying predictors and correlates of weight loss and maintenance: implications for weight control therapies based on behaviour change. Obes Rev.

[19] Kolb H., Stumvoll M., Kramer W., Kempf K., Martin S. (2018). Insulin translates unfavourable lifestyle into obesity. BMC Med.

[20] Varkevisser R.D.M., van Stralen M.M., Kroeze W., Ket J.C.F., Steenhuis I.H.M. (2019). Determinants of weight loss maintenance: a systematic review. Obes Rev.

[21] Chopra S., Malhotra A., Ranjan P. (2021). Predictors of successful weight loss outcomes amongst individuals with obesity undergoing lifestyle interventions: a systematic review. Obes Rev.

[22] Desai M. (2020). Recruitment and retention of participants in clinical studies: critical issues and challenges. Perspect Clin Res.

[23] Wasfi R., Poirier Stephens Z., Sones M. (2021). Recruiting participants for population health intervention research: effectiveness and costs of recruitment methods for a cohort study. J Med Internet Res.

[24] Sonne-Holm S., Sørensen T.I., Jensen G., Schnohr P. (1989). Influence of fatness, intelligence, education and sociodemographic factors on response rate in a health survey. J Epidemiol Community Health.

[25] Tchkonia T., Thomou T., Zhu Y. (2013). Mechanisms and metabolic implications of regional differences among fat depots. Cell Metab.

[26] Andersson D.P., Eriksson Hogling D., Thorell A. (2014). Changes in subcutaneous fat cell volume and insulin sensitivity after weight loss. Diabetes Care.

[27] Masood B., Moorthy M. (2023). Causes of obesity: a review. Clin Med (Lond).

[28] Arner P., Bernard S., Salehpour M. (2011). Dynamics of human adipose lipid turnover in health and metabolic disease. Nature.

[29] Petrus P., Mejhert N., Corrales P. (2018). Transforming growth factor-β3 regulates adipocyte number in subcutaneous white adipose tissue. Cell Rep.

[30] Lönn M., Mehlig K., Bengtsson C., Lissner L. (2010). Adipocyte size predicts incidence of type 2 diabetes in women. FASEB J.

[31] Weyer C., Foley J.E., Bogardus C. (2000). Enlarged subcutaneous abdominal adipocyte size, but not obesity itself, predicts type II diabetes independent of insulin resistance. Diabetologia.

[32] Eriksson-Hogling D., Andersson D.P., Bäckdahl J. (2015). Adipose tissue morphology predicts improved insulin sensitivity following moderate or pronounced weight loss. Int J Obes (Lond).

