# Supplementary material for: Adipose cellularity as a measurement of long-term changes in body weight: a Swedish cohort study spanning 1988–2016
Source: eClinicalMedicine. 2025 Mar 29;82:103165. doi: 10.1016/j.eclinm.2025.103165 (PMC11997358; doi:10.1016/j.eclinm.2025.103165)
Supplement: Fig. S1 — Flow chart for key measures. In total 1014 subjects were initially investigated. For subcutaneous (sc) number data were missed on 49 subjects and for body weight/body mass index (BMI) data were not recorded for 3 subjects. Visceral fat cell size was recorded for a fraction of the initially investigated subjects. On all of them we had data for body weight and/or BMI. A second investigation was performed on 281 subjects. On all of them we had follow up data on body weight and BMI as well as initial data on sc fat cell size. Data on sc fat cell number was missing on 7 subjects but on all remaining ones we had initial and follow up data on body weight/BMI. For the subgroup with initial data on visceral fat cell size we had body weight/BMI data at initial–and follow-up investigation. [file mmc1.pptx]

## Slide 1
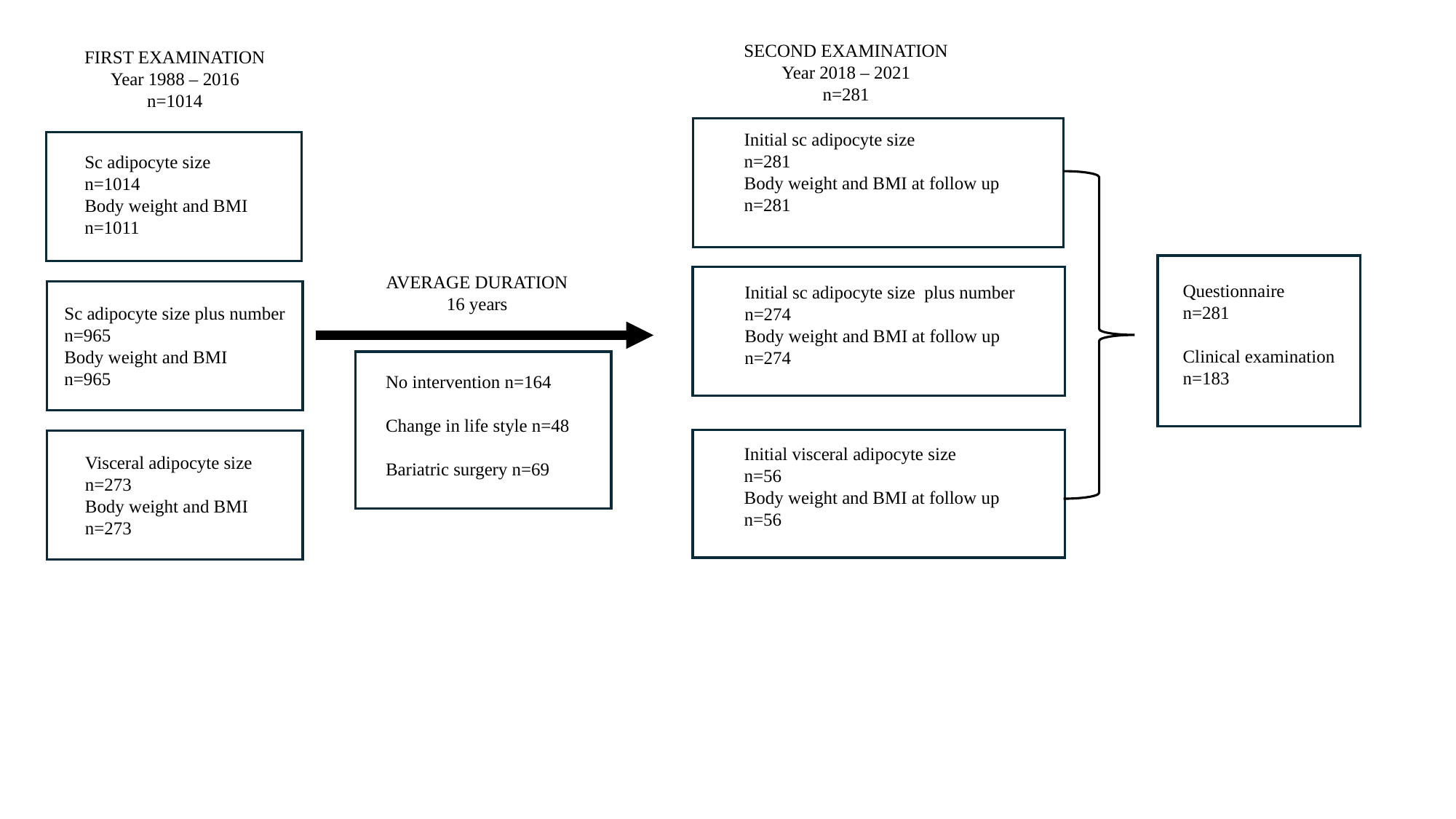

SECOND EXAMINATION
Year 2018 – 2021
n=281
Initial sc adipocyte size
n=281
Body weight and BMI at follow up
n=281
Initial sc adipocyte size plus number
n=274
Body weight and BMI at follow up
n=274
Initial visceral adipocyte size
n=56
Body weight and BMI at follow up
n=56
FIRST EXAMINATION
Year 1988 – 2016
n=1014
Sc adipocyte size
n=1014
Body weight and BMI
n=1011
Sc adipocyte size plus number
n=965
Body weight and BMI
n=965
Visceral adipocyte size
n=273
Body weight and BMI
n=273
Questionnaire
n=281
Clinical examination
n=183
AVERAGE DURATION
16 years
No intervention n=164
Change in life style n=48
Bariatric surgery n=69
