# Supplementary material for: Adipose cellularity as a measurement of long-term changes in body weight: a Swedish cohort study spanning 1988–2016
Source: eClinicalMedicine. 2025 Mar 29;82:103165. doi: 10.1016/j.eclinm.2025.103165 (PMC11997358; doi:10.1016/j.eclinm.2025.103165)
Supplement: Table S1 [file mmc2.docx]

| Group | Adipocyte size | | | Adipocyte number | | |
| --- | --- | --- | --- | --- | --- | --- |
|  | Number of patients | Beta coefficient | p-value | Number of patients | Beta coefficient | p-value |
| No obesity * | 136 | -0·14 | 0·095 | 130 | 0·13 | 0·13 |
| Obesity | 146 | -0·37 | <0·0001 | 144 | -0·36 | <0·0001 |
| Sedentary | 71 | -0·46 | <0·0001 | 71 | -0·28 | 0·002 |
| Physically active | 175 | -0·55 | <0·0001 | 169 | -0·39 | <0·0001 |
| Males | 86 | -0·30 | 0·0057 | 82 | -0·26 | 0·021 |
| Females | 196 | -0·56 | <0·0001 | 192 | -0·40 | <0·0001 |
| Using nicotine | 48 | -0·61 | <0·0001 | 47 | -040 | 0·005 |
| Not using nicotine | 229 | -0·48 | <0·0001 | 223 | -0·39 | <0·0001 |

Table S1. The relationship between abdominal subcutaneous adipocyte size (picolitres) or number and changes in body weight (kg) over time in in subgroups. Linear regression was used. Negative beta coefficients indicate that the relationship with body fat changes is inverse. *When one outlying subject reporting 200 kg body weight at follow-up using home scale weighing was excluded the values regarding adipocyte volume were beta coefficient=-0.20 and p=0.021. Exclusion of this subject did not improve the relationship between adipocyte number and changes in body weight (beta coefficient=-0.07 and p=0.46).
